# High-Resolution and Large-Detection-Range Virtual Antenna Array for Automotive Radar Applications

**DOI:** 10.3390/s21051702

**Published:** 2021-03-02

**Authors:** Haythem Abdullah, Mohamed Mabrouk, Ahmed Abd-Elnaby Kabeel, Amr Hussein

**Affiliations:** 1 Microwave Engineering Department, Electronics Research Institute, El-Nozha Elgededa, Cairo 11843, Egypt; 2 Electronic and Communication Department, Faculty of Engineering, Alexandria University, Alexandria 21544, Egypt; mmabr084@uottawa.ca; 3 Electronics and Communications Engineering Department, High Institute of Engineering and Technology, New Damietta 34517, Egypt; ahmed.kabeel@ndeti.edu.eg; 4 Electronics and Electrical Communications Engineering Department, Faculty of Engineering, Tanta University, Tanta 31527, Egypt; amr-abdallah@f-eng.tanta.edu.eg

**Keywords:** automotive radar, long-range radar, medium-range radar, linear antenna array, virtual antenna array

## Abstract

Collision avoidance and autonomous control of vehicles have become essential needs for providing a high-quality and safe life. This paper introduces a new generic scheme for a virtual antenna array (VAA) and its application in a train collision-avoidance system (TCAS). The proposed TCAS shall have the capability of identifying the range and angle of an object in front of a moving train and provide the required alerts. Thereby, a new virtual array distribution for both the transmitting and the receiving antenna arrays is introduced to get a long-range object detection and high-resolution multi-input multi-output (MIMO) system. This can be accomplished because the VAA radiation pattern is the multiplication of the radiation patterns for both the transmitting and receiving antenna arrays, which is different than each one of them alone. In this work, the VAA is utilized in radar systems in which the radar range depends on the multiplication of the gain of the transmitting and receiving antennas. So, we introduce a new scheme for the general design of VAA-based radars. A prototype for the antenna system was fixed on a of Texas Instruments platform for the cascading radar. One of the main problems of the VAA is the loss of radiated power in undesired directions, which affects the maximum detection range in beamforming systems and degrades the diversity gain in MIMO applications. These issues have been solved by the introduction of the practical implementation of a proposed high-gain, low side lobe level VAA system for automotive radar that is based on the integration of four AWR1243 RF chips operating in a frequency range of 76 GHz to 81 GHz. It was implemented using low-power 45 nm (TI) RFCMOS technology. The measured gain of the realized VAA was 47.2 dBi, which was 1.815 times higher than that of the Texas instrumentation linear frequency modulated continuous wave (TI’ LFMCW) radar, which was 26 dBi. The proposed VAA saved 45% of the required implementation area compared to the TI’ LFMCW antenna array. The VAA system was fabricated and tested in an anechoic chamber, and it was found that the simulated and measured patterns of the proposed VAA were highly matched in terms of half-power beamwidth and side lobe level.

## 1. Introduction

Recently, radars and multi-input multi-output (MIMO) radars have been extensively used as contactless sensors in critical applications such as vital-sign detection and human localization [1], imaging systems [2], and static and dynamic monitoring of bridges [3]. In [1], the authors introduced an extensive review of the utilization of MIMO radars in biomedical applications, such as monitoring the health conditions of humans and detecting their presence in different environments. They also handled different array configurations (either one- or two-dimensional arrays), and discussed their effects on the radar-received signal-to-noise ratio, radar detection range, angular resolution for the angle of arrival (AoA) estimation, localization of objects, and accuracy of signal detection. In [2], a calibration method for a MIMO radar-based high-resolution imaging system was introduced to mitigate undesired antenna effects such as proximity coupling between two neighboring antennas, the imperfect reflection coefficient of the antenna $S_{11}$, and direction of arrival (DoA) dependency on the antenna type, which result in position shifts of the detected objects. In [3], the authors introduced a multi-monostatic interferometric (2 × 2) MIMO radar system to detect the displacement vector of large bridge structures. The experimental results showed the effectiveness of their proposed system.

In recent years, 77 GHz automotive radars have become popular and have drawn the interest of many researchers around the world, particularly to achieve a longer detection range and better range resolution. For this purpose, the authors in [4] introduced a two-stage 0.1 µm AlGaAs/InGaAs/GaAs PHEMT MMIC chip 77 GHz power amplifier that is ideal for automotive radar applications. The amplifier has a small size of $\left(3\times 2\right){\;\mathrm{mm}}^{2}$ and provides a 10 dB signal gain with a maximum output power of 140 mW. For electronic phased array radars, millimeter-wave beam steering is a critical issue that requires a special treatment to mitigate the distortions arising from the unbalanced feeding amplitudes among the delay lines of the feeding network. For this purpose, the authors in [5,6] introduced a passive millimeter-wave, distortion-free beam-steering solution with analog resolution in liquid-crystal planar delay-line technology and a novel automatic attenuator with an ultra-fast transient response time, respectively.

On the other hand, the virtual antenna array (VAA) concept has emerged as a promising beamforming technique in high-performance radar systems that are equipped with separate transmit and receive antenna arrays. VAA allows the proper utilization of the transmitter array (TA) and the receiver array (RA) for the synthesis of wide varieties of highly efficient and cost-effective radiation patterns that are fully dependent on the TA and RA radiation patterns. However, the synthesized patterns may have distinct characteristics compared to that of the individual patterns of the transmitter and receiver arrays. The implementation of a VAA is not restricted to a specific design scheme. Recently, many VAA beamforming schemes have emerged for performance enhancement of traditional radar systems, multi-input multi-output (MIMO) radars, and automotive radars [7,8,9,10,11,12,13,14,15,16,17,18,19,20,21].

In [7], a virtual array beamforming (VAB) technique for generating simultaneous multiple transmit/receive beams at different directions was introduced. In this technique, each antenna element from the utilized L-elements linear antenna array transmits a distinct orthogonal waveform. At the receiver, the received waveforms at each antenna element are separated using a bank of L-matched filters. The detected L-versions of each distinct waveform at the L-similar matched filters are weighted and fed to a delay and sum beamformer to generate a virtual beam. The distinct virtual beam can be steered to any direction by changing the weighting vector of the L-similar matched filters without changing the excitation coefficients of the transmitting antenna elements, as in the case of passive phased antenna arrays. However, the implementation of this VAB system requires a large number (L×L) of matched filters and L delay and sum beamformers of high cost.

In [8], an effective VAA beamforming technique was investigated for converting a single parabolic reflector antenna into a VAA radiating in the broadside direction. Its main idea was based on generating a number of displaced phase center locations or illumination regions on the parabolic aperture that are displaced from its physical center. The phase center locations can be changed electronically by controlling the parameters of the feeder modes such as the number of operating modes, amplitude and phase of mode excitation, mode polarization, and mode orientation. These illumination regions form an array of virtual elements with different locations and excitations.

In [9], a VAA beamforming technique was introduced for pattern null broadening. The array excitations are derived from the modified covariance matrix of interference plus noise that is defined as the Kronecker product of the covariance matrix and its conjugate. The Kronecker product maximizes the large eigenvalues of the modified matrix and hence produces deeper nulls. In [10], a virtual large-scale array beamforming technique based on the measured patterns of small-size subantenna arrays was introduced. The virtual large-scale arrays were formed by repositioning or shifting the small-size subarray M-times in a single direction or two directions while maintaining the same element spacing and the existence of nonoverlapping elements. This technique has been applied in uniform linear antenna arrays (ULAs), uniform rectangular antenna arrays (URAs), and uniform circular antenna arrays (UCAs). Along these lines, a large-scale virtual uniform rectangular array for millimeter-wave channel characterization was introduced in [11]. The utilization of large-scale VAAs provided massive sampling, which enabled high spatial resolution in channel sounding. The VAA concept also has gained a lot of attention in biomedical microwave imaging applications, as the accuracy of imaging is highly dependent on the number of spatially spaced real or virtual antenna elements, which indeed increases the recorded signal samples and produces pure images [12]. In the field of the direction of arrival (DOA) estimation, the VAA utilization significantly achieved higher detection accuracy and higher resolution, and increased the number of detectable sources [13,14,15,16].

For MIMO synthetic aperture radars (SAR), several VAA configurations have been introduced to improve the performance of remote-sensing systems. Taking into consideration the phase differences arising from the transmitting antenna elements with the phase differences caused by the receiving antenna elements, new sets of virtual antenna array steering vectors can be generated by optimizing the designed antenna array positions [17].

The VAA concept is not confined to linear and rectangular array configurations only, but also has been achieved for hexagonal array configurations. In this context, a 49-element virtual hexagonal antenna array was formed from two aligned 7-element hexagonal transmitting and 7-element hexagonal receiving arrays, as introduced in [18]. The simulations demonstrated that the hexagonal-shaped VAA achieved better side lobe suppression and improved angular resolution compared to the rectangular-shaped VAA. In [19], a new LFMCW-MIMO airborne radar system applying a linear frequency modulated continuous wave (LFMCW) signal and virtual MIMO was introduced and analyzed. In [20], a combined MIMO processing with an array extrapolation method based on Burg’s algorithm was introduced for the enhancement of the angular resolution of MIMO radars. The Burg’s algorithm was utilized to compensate for the missing virtual antenna elements caused by the breakdown of some of the real receive antenna elements. In [21], a statistical analysis of radar electronics faults and positioning phase error effects on the peak side lobe level (PSLL) of the virtual array-based MIMO radar system was introduced. It was concluded that the nonuniform virtual array provided better phase tolerance compared to the uniform virtual arrays. In [22], a novel VAA synthesis technique based on the method of moments (MoM) and the genetic algorithm (GA) was introduced for the synthesis of planar antenna arrays (PAAs) in radar systems. This technique mimicked PAAs by using two orthogonal linear antenna arrays (LAAs) whose excitation coefficients were optimized to achieve a pattern highly matched to that of the desired PAA.

In this paper, the drawbacks of the VAA are mitigated to a large extent while retaining the main merits of low cost and optimum number of resources. In our case, we considered a monostatic radar equipped with two separate LAAs. The contribution of this research focuses on introducing a new generic scheme for synthesizing a radar total transceiver beam in a flexible and cost-effective procedure. The transmitter and the receiver antennas may be distributed in different configurations, keeping the resulting VAA beams with nearly similar beams. This allows flexibility in synthesizing the required VAA beam according to the available transmitter sources, the number of receivers, and the available space for the transmitting and the receiving antennas. Along these lines, a different number of VAA configurations with different radiation characteristics can be realized based on the design of the utilized LAAs and the relative alignment between them. Furthermore, practical implementation and testing of a radar transceiver antenna to be applicable in a low-speed train collision-avoidance system are introduced. The proposed radar system is based on the integration of four AWR1243 RF chips operating in a frequency range of 76 GHz to 81 GHz. It was implemented using low-power 45 nm (TI) RFCMOS technology. The fabricated VAA system achieved a 47.2 dBi gain, which was 1.815 times higher than the gain of the TI’ LFMCW radar, which was 26 dBi. Furthermore, the proposed VAA saved 45% of the required implementation area compared to the TI’ LFMCW antenna array. In addition, the fabricated VAA had a fast-decaying side lobe level (SLL) with ${\mathrm{SLL}}_{max}=-13\;\mathrm{dB}$ and ${\mathrm{SLL}}_{min}\approx -55\;\mathrm{dB}$. These features provided a large detection range with high resolution. It is worth mentioning that the simulations were performed using MATLAB R2018a and verified using a computer simulation studio (CST) microwave studio software package. The proposed VAA system was fabricated on a RO3003 substrate of thickness h=0.13 mm and had a dielectric constant $\varepsilon_{r}=3$, which was tested by Roger in the frequency range of 8 to 40 GHz and tangent loss of 0.001, which was measured at 10 GHz 23 °C.

## 2. Virtual Antenna Array Concept

**Figure 1:**
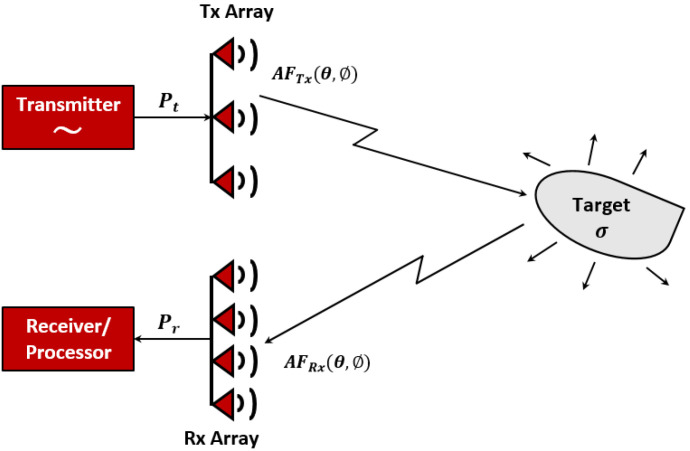
A schematic diagram of the general radar system.

In radar systems, one or two separate antenna arrays are used for transmitting and receiving functions, as illustrated in Figure 1. If the transmitting and receiving antennas are collocated, the system is called monostatic radar. Otherwise, it is called bistatic radar. The monostatic radar transmitter and receiver antennas can be arranged to apply the VAA concept. In such radars, the received signal strength depends on the radiation pattern that is constituted from the multiplication of the radiation patterns for the transmitting and receiving antenna arrays [23].

The advantages of such radar systems are: (i) If the transmitter and receiver arrays have the same side lobe levels (SLLs), the SLL of the resulting pattern is the square of the individual SLL value, which can be calculated as twice the negative value of the individual SLL in decibels. Nevertheless, if the transmitter and receiver arrays have different SLLs, the resultant SLL is sum of the SLLs of both the transmitter and receiver in decibels. (ii) The radiated power is concentrated in the direction of the main beam, while the received power is enhanced by the maximum gain value of the receiving antenna array.

These features are useful for military radars where the interference with any neighboring source represents a danger to the radar operations. Nevertheless, in automotive radars, the interference is not the main concern, since the vehicles are moving so that most interferences may occur momentarily. Therefore, the interest of designers in automotive radars focuses on the pattern beamwidth with acceptable SLL. Furthermore, the cost of the radar depends mainly on the number of transmitters, voltage-controlled phase shifters, and voltage-controlled amplifiers or attenuators. From these points, the concept of VAAs appears where one can achieve a cost-effective radar with a minimum number of resources that achieve the required beamwidth as the traditional radars. In this research, we introduce a proposed scheme for the transmitter array to enhance the SLL to large extent, but the immunity to interference depends mainly on the receiving array, which is kept to its standard value.

## 3. Proposed VAA Analysis

**Figure 2:**
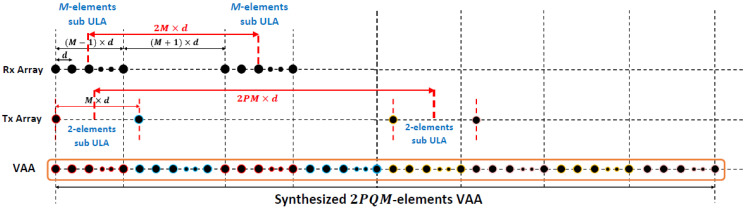
Proposed general VAA schematic diagram.

Taking the proposed general scenario shown in Figure 2, where the receiver antenna array is made of P subarrays, each consisting of an M-elements uniform linear array (ULA). The spacing between the elements of the receiving subarrays is d, while the distance from the centers of any two successive receive subarrays is 2M×d. The transmitter array is made of Q subarrays, each consisting of a 2-element ULA with M×d element spacing. The spacing from the two centers of any two successive transmit subarrays is 2PM×d.

In this case, the transmitter and receiver array factors are $AF_{Tx}\left(\theta ,\emptyset \right)$ and $AF_{Rx}\left(\theta ,\emptyset \right)$, respectively. The receiver array factor, $AF_{Rx}\left(\theta ,\emptyset \right),$ is calculated by multiplying the array factor of the *M*-elements sub-ULA ($AF_{Rx-sub})$ by the array factor of the P  array of the M-elements subarrays ($AF_{Rx-Parray})$ where $AF_{Rx-sub}$, $AF_{Rx-Parray}$ and $AF_{Rx}\left(\theta ,\emptyset \right)$ are calculated according to Equations (1) and (2), respectively.

(1) $$AF_{Rx-sub}\left(\theta ,\emptyset \right)=\overset{M-1}{\underset{m=0}{\sum }}e^{jkmd\sin\theta \cos\emptyset }$$

(2) $$AF_{Rx-Parray}\left(\theta ,\emptyset \right)=\overset{P-1}{\underset{p=0}{\sum }}e^{2jkpMd\sin\theta \cos\emptyset }$$

(3) $$AF_{Rx}\left(\theta ,\emptyset \right)=AF_{Rx-sub}\left(\theta ,\emptyset \right)\times AF_{Rx-Parray}\left(\theta ,\emptyset \right)$$

Similarly, the transmitter array factor $AF_{Tx}\left(\theta ,\emptyset \right)$ is calculated by the multiplication of the 2-element sub-ULA array factor ($AF_{Tx-sub}\left(\theta ,\emptyset \right)$) by the array factor of the Q array of the 2-element subarrays, $AF_{Tx-Qarray}$, according to Equations (4)–(6), respectively.

(4) $$AF_{Tx-sub}\left(\theta ,\emptyset \right)=\overset{1}{\underset{l=0}{\sum }}e^{jklMd\sin\theta \cos\emptyset }$$

(5) $$AF_{Tx-Qarray}\left(\theta ,\emptyset \right)=\overset{Q-1}{\underset{q=0}{\sum }}e^{2jkqpMd\sin\theta \cos\emptyset }$$

(6) $$AF_{Tx}\left(\theta ,\emptyset \right)=AF_{Tx-sub}\left(\theta ,\emptyset \right)\times AF_{Tx-Qarray}\left(\theta ,\emptyset \right)$$

The virtual array factor ($AF_{VA}\left(\theta ,\emptyset \right))$ is the multiplication of the transmitter and receiver array factors according to Equation (7).

(7) $$AF_{VA}\left(\theta ,\emptyset \right)=\;AF_{Tx}\left(\theta ,\emptyset \right)\times AF_{Rx}\left(\theta ,\emptyset \right)$$

By substituting from Equation (1) to Equation (6) into Equation (7) and by some mathematical manipulations, the virtual array factor is calculated as the following:

(8) $$AF_{VA}\left(\theta ,\emptyset \right)=\overset{N-1}{\underset{n=0}{\sum }}e^{jknd\sin\theta \cos\emptyset }$$

(9) N=2PQM

where N is the equivalent number of antenna elements of the synthesized VAA, 2 is the number of transmit subarray elements, P is the repetition number of receiver subarrays, Q is the number of repetitions for these subarrays, and M is the number of receive subarray elements.

Accordingly, Equation (8) reveals that the synthesized VAA is equivalent to an N-element ULA with uniform element spacing d.

## 4. Proposed VAA Configurations

### 4.1. Synthesis of 40-Elements VAA

**Figure 3:**
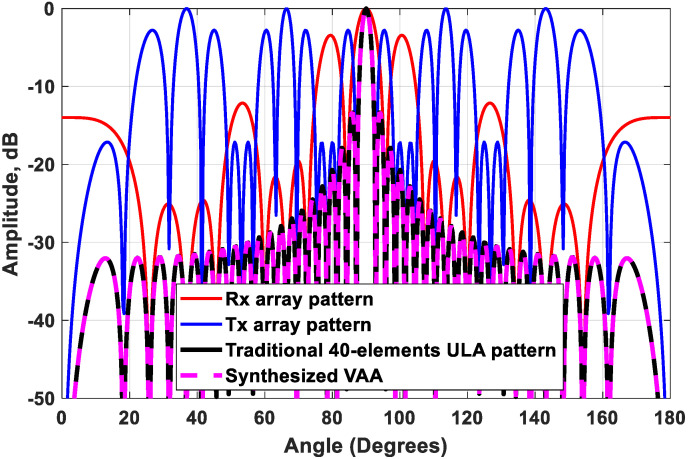
The synthesized N=40-element VAA pattern for M=5, Q=2, P=2, and d=λ/2 compared to the traditional 40-element ULA pattern.

As an extension of the first test case, a nonoverlapping 40-element VAA pattern can be synthesized following the general array design and alignment criteria shown in Figure 2. The receiving array has a repetition number of Q=2 for M=5-element ULAs with element spacing d=λ/2, and their centers are separated by a distance 2M×d=10d. While a 2-element sub ULA with element spacing M×d=5d is added to the transmitter antenna array in this case, the repetition number will be P=2. The separation distance between the centers of the two 2-element sub-ULAs equals 2PM×d=2×2×5×d=20d. As a result, the synthesized VAA array factor can be expressed in the form of a traditional ULA consisting of N=2PQM=2×2×2×5=40 antenna elements with uniform element spacing d=λ/2. The synthesized VAA pattern perfectly coincides with the traditional 40-element ULA pattern, as shown in Figure 3.

### 4.2. Synthesis of High-Gain, Low-SLL VAA

**Figure 4:**
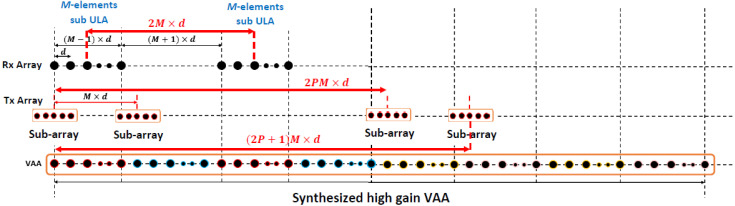
The synthesized high-gain VAA using four subarrays at the transmitting side.

In this case, a high-gain VAA pattern is synthesized following the array design and alignment criteria shown in Figure 4. For clarification, let M=8, Q=2, P=2, and d=λ⁄2. Each antenna element of the two transmit sub-ULAs is replaced by a subantenna array consisting of L elements, with the center of each subarray being the substituted antenna element, as shown in Figure 4. The array factor of the subarray is given by:

(10) $$AF_{SUB}\left(\theta ,\emptyset \right)=\overset{L-1}{\underset{l=0}{\sum }}a_{l}e^{jkld_{s}\sin\theta \cos\emptyset }$$

where $a_{l}$ is the excitation coefficient of the $l^{th}$ antenna element of the subarray and $d_{s}$ is the element spacing of the subarray. Accordingly, the equivalent virtual array factor can be written as:

(11) $$AF_{VA}\left(\theta ,\emptyset \right)=\left\{AF_{SUB}\left(\theta ,\emptyset \right)\times AF_{Tx}\left(\theta ,\emptyset \right)\right\}\times AF_{Rx}\left(\theta ,\emptyset \right)$$

**Figure 5:**
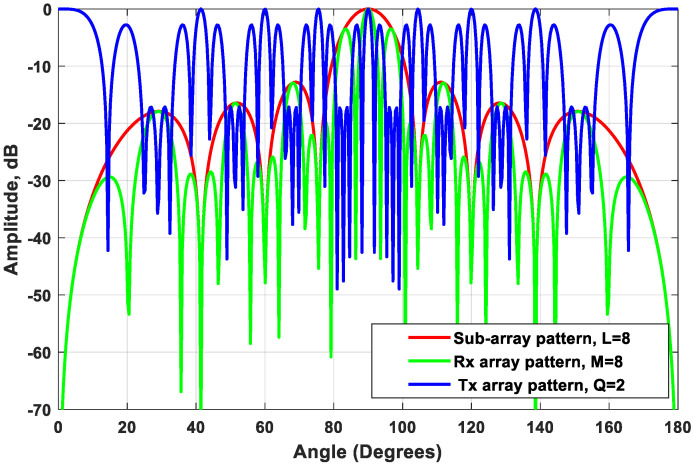
The arrays patterns of the receiver array, transmitter array, and the subarray for M=8, Q=2, P=2, L=8, and $d_{s}=d=\lambda ⁄2$.

**Figure 6:**
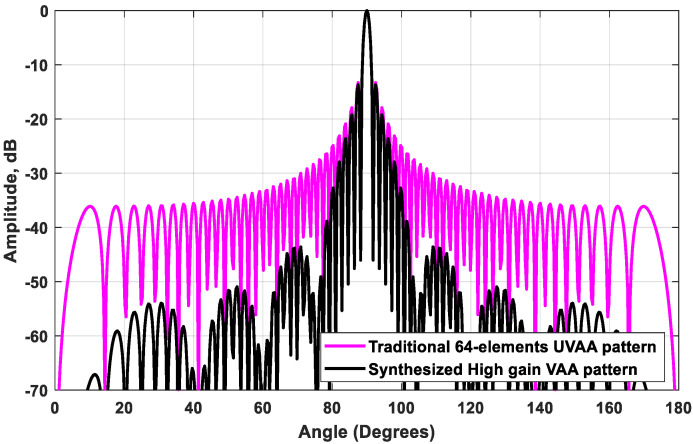
The synthesized high-gain VAA pattern for M=8, Q=2, L=8, and $d_{s}=d=\lambda /2$, compared to the traditional 64-element UVAA pattern.

In this case, the outcome of the proposed beamforming technique is a high-gain VAA with fast-decaying side lobes. The array radiation patterns of the new configuration are plotted in Figure 5. When analyzing each radiation pattern separately, it was noticed that the receiving array pattern had a very high side lobe up to −3 dB, while 8 grating lobes appeared in the radiation pattern of the transmitting array because of the distance between the elements of the array, which exceeds the multiples of the wavelength. However, when applying the proposed VAA beamforming and multiplying the three patterns as in (11), a high-gain VAA pattern with fast-decaying side lobes was obtained completely without any grating lobes, as shown in Figure 6. It was clear that the radiation pattern of the synthesized VAA array using four subarrays had much lower side lobes compared to the traditional 64-element uniform virtual antenna array (UVAA) that was synthesized using only Q=2 sub-2-element ULAs.

The subarray was chosen to have the same number of elements as the receiving array. This allowed the nulls of the subarray to occur at the locations of the transmitter grating lobes, as illustrated in Figure 5. Figure 6 illustrates the multiplication of the subarray array factor by the transmitter array factor, where it was noticed that the locations of the nulls occurred clearly at the angles of the previous grating lobes. Further improvement of the SLL could be achieved by the proper selection of the subarray elements feeding and spacing.

### 4.3. Practical Implementation of the VAA for Automotive Radars

A. Problem formulation

The implementation of the proposed automotive radar system was based on the integration of four AWR1243 RF chips. The AWR1243 is a single-chip standalone frequency-modulated continuous-wave (FMCW) transceiver system that implements the automotive radar sensors in the range of 76 GHz to 81 GHz. It was implemented using low-power 45 nm Texas Instruments (TI) RFCMOS technology. Each chip had three transmitters and four receivers. Therefore, when using four AWR1243 chips, we had only 3×4=12 transmitter sources While the array configuration in Figure 4 had two M=8-element receiver ULAs and four L=8-element transmitter subarrays for a total of 32 transmitter antenna elements, which requires 32 transmitter sources, this imposed a great difficulty in implementing the proposed VAA system. In the next section, the solution to this problem is introduced in detail, ending with a complete operating system.

B. Proposed VAA design considerations

**Figure 7:**
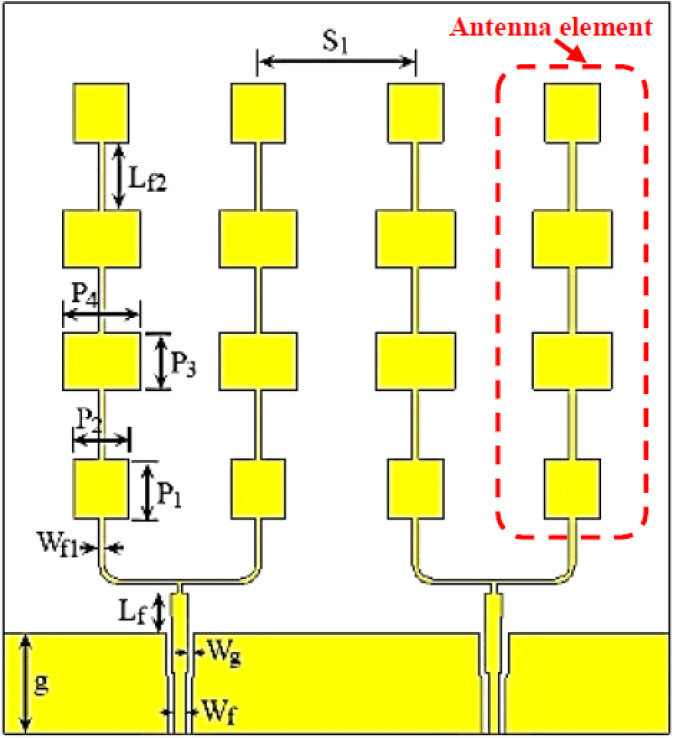
Four elements of the transmitter subarray where each of the two elements share a common feed via a T-junction power divider.

To mitigate the problem of the limited number of transmitter sources of the four AWR1243 integrated chips, the number of elements of the L=8-element subarray could be reduced to only L=6 elements while maintaining the same array size $A_{s}$ so as not to affect the half-power beamwidth (HPBW) of the pattern. This can be achieved by increasing the element spacing to $A_{s}/6$ instead of $A_{s}/8\;$and keeping the same elements excitations. Accordingly, we had 24 transmit antennas, which required 24 transmitting sources. In order to reduce this number of transmitting sources to the available 12 sources, every two neighboring elements were designed to share a common feed via a T-junction power divider, as shown in Figure 7. Each antenna element in Figure 7 consisted of four cascaded microstrip patch antennas. The dimensions of the rectangular patch antenna elements were designed to operate at 77 GHz. The shown four elements of the subarray were optimized to get good matching at the desired frequency band. Then a T-junction power divider was added to each two neighboring elements to be fed from a single source. The matching was obtained by optimizing the widths and lengths of the feeding transmission lines. The values of the antenna parameters shown in Figure 7 are listed in Table 1. The element was implemented on a RO3003 substrate with dielectric constant $\varepsilon_{r}=3$, tangent loss of 0.001, and thickness h=0.13 mm.

**Figure 8:**
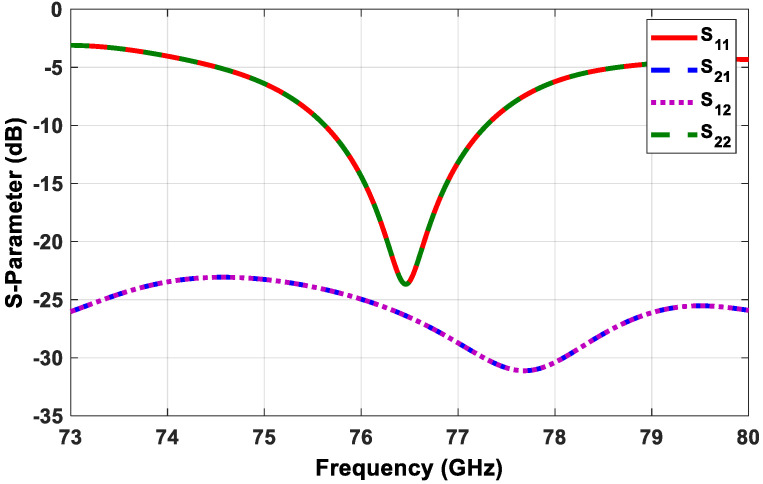
Simulated S-parameters of the proposed transmitter subarray configuration.

For the sake of brevity, the design steps for the transmitter subarray elements and the scattering (S) parameters of the single antenna were omitted. Figure 8 demonstrates the simulated S-parameters of the proposed transmitter antenna configuration shown in Figure 7.

There are several parameters that measure MIMO parameters, such as the envelop correlation coefficient (*ECC*), the diversity gain (DG), and the total active reflection coefficient (*TARC*).

**Figure 9:**
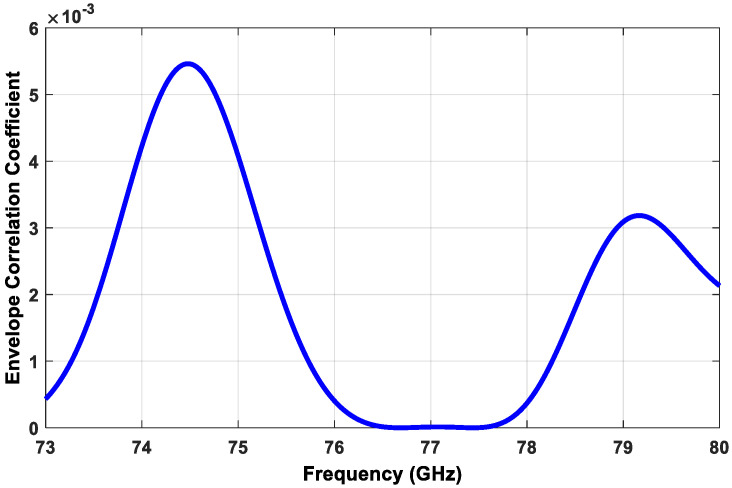
Simulated envelope correlation coefficient of the proposed transmitter subarray configuration.

The *ECC* measures the similarity between the received signals of two consequent antennas. The $ECC_{mn}$ is calculated according to Equation (12)

(12) $$ECC_{mn}=\frac{{\left|\int \;\int_{0}^{4\pi }\left[{\vec{F}}_{m}\left(\theta ,\emptyset \right)\times {\vec{F}}_{n}\left(\theta ,\emptyset \right)\right]d\Omega \right|}^{2}}{\int \;\int_{0}^{4\pi }{\left|{\vec{F}}_{m}\left(\theta ,\emptyset \right)\right|}^{2}d\Omega \int \;\int_{0}^{4\pi }{\left|{\vec{F}}_{n}\left(\theta ,\emptyset \right)\right|}^{2}d\Omega }$$

where ${\vec{F}}_{m}\left(\theta ,\emptyset \right)$ and ${\vec{F}}_{n}\left(\theta ,\emptyset \right)$ are the antenna radiation patterns of the $m^{th}$ and $n^{th}$ antennas, respectively, and m, n = 1,2,3,4, …etc. The simulated *ECC* is shown in Figure 9; its maximum value did not exceed 0.006, which is considered a very good result according to the recommended value of below 0.3 in literature [24,25], which meant a very low correlation between the MIMO antennas.

The second parameter is the diversity gain (DG), which can be expressed as Equation (13) [26,27].

(13) $$\mathrm{DG}=10\times \sqrt{1-ECC^{2}}$$

**Figure 10:**
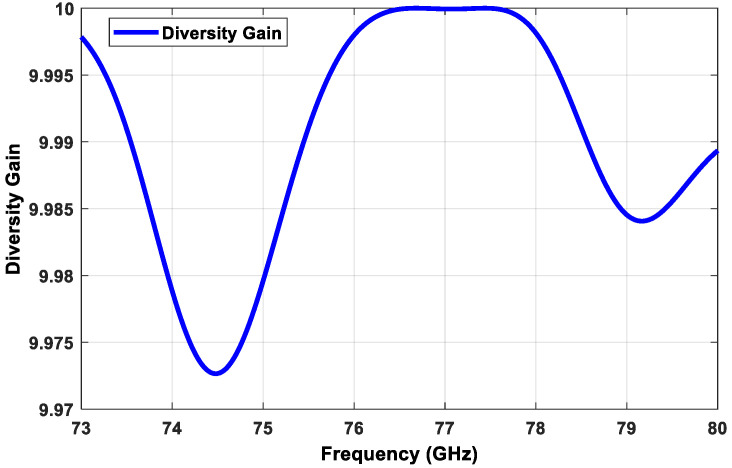
Simulated DG of the proposed transmitter subarray configuration.

Figure 10 shows the simulated DG of the proposed transmitter subarray configuration, which approached the optimal value of 10 within the desired frequency band.

**Figure 11:**
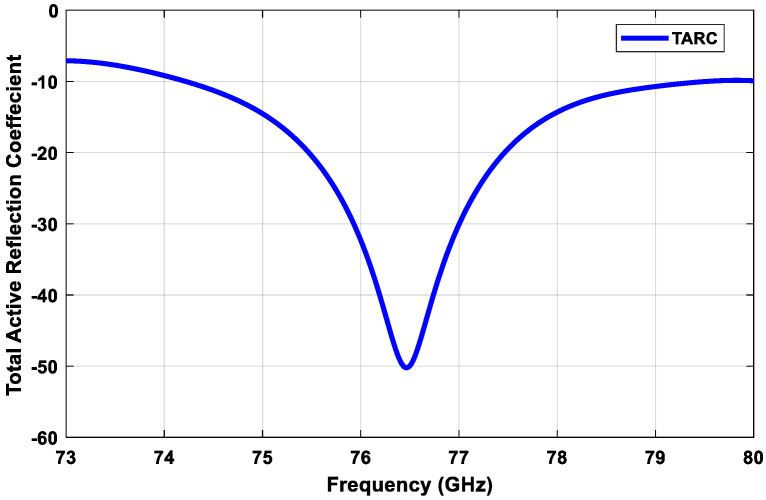
Simulated *TARC* calculations for the proposed transmitter subarray configuration.

The third parameter that shows the coupling among ports is *TARC*. *TARC* means the ratio of the square root of the total reflected power divided by the square root of the full incident power [28]. *TARC* means the return loss of the overall MIMO antenna elements. *TARC* is calculated using the two antennas’ scattering parameters, as in Equation (14) [29]:

(14) $$TARC=\sqrt{\frac{{\left|s_{11}+s_{12}e^{j\theta }\right|}^{2}+{\left|s_{21}+s_{22}e^{j\theta }\right|}^{2}}{2}}$$

where θ is a variable angle that is correlated to the phase of the excitation signal. To obtain the average *TARC*, it utilizes the random phase-excitation vectors. The MIMO channel is expected to be Gaussian with a multipath spread in the propagation channel [29]. *TARC* is considered at each random phase between 0 and 180. Figure 11 displays the average value of the *TARC* of the MIMO antennas. It is noted that the simulated *TARC* ranged between −10 dB and −50 dB within the operating band, which were considered accepted values.

**Figure 12:**
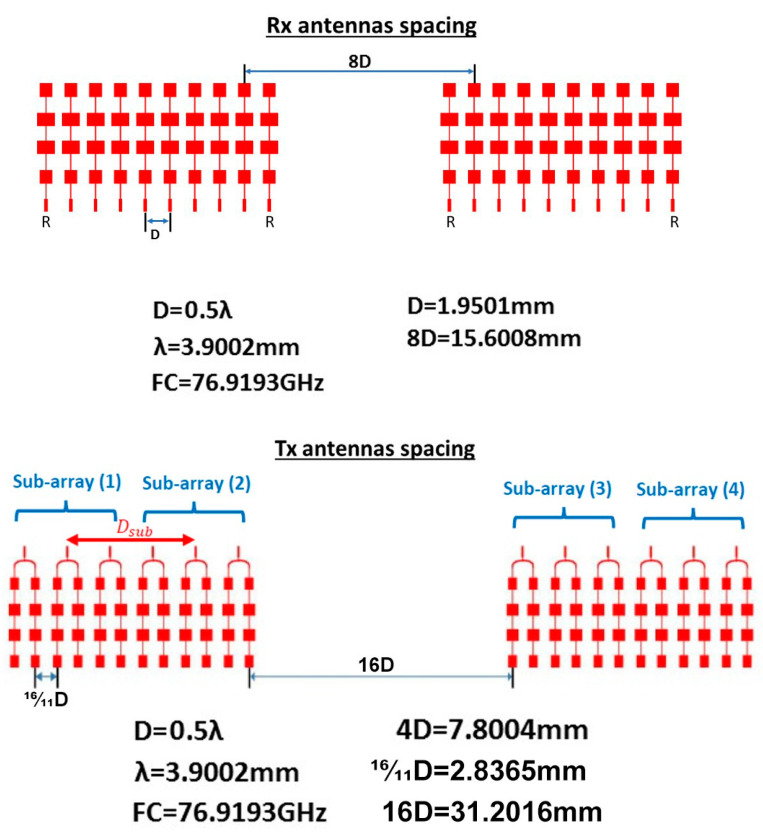
Proposed array configurations for the LFMCW radar (**a**) transmitter array and (**b**) receiver array.

**Figure 13:**
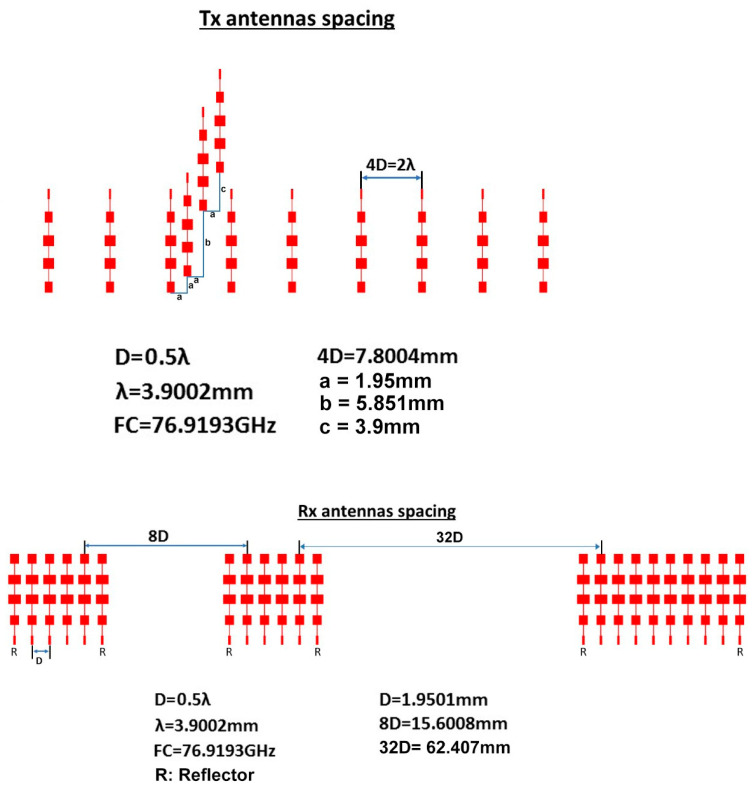
Antenna array configurations of the traditional TI’ LFMCW radar introduced in [31]: (**a**) transmitter array and (**b**) receiver array.

**Figure 14:**
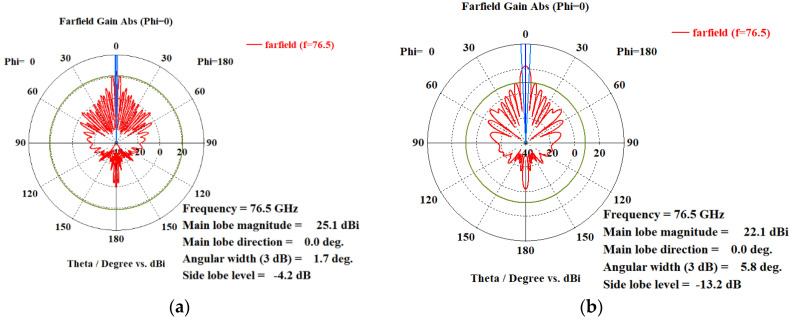

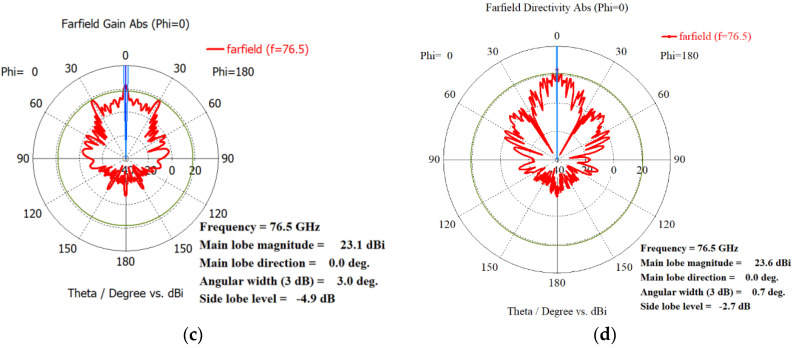
Polar radiation patterns of: (**a**) transmitter array of the proposed VAA, (**b**) receiver array of the proposed VAA, (**c**) transmitter array of the traditional TI’ LFMCW radar, and (**d**) receiver array of the traditional TI’ LFMCW radar.

Using the proposed array distribution and the proposed antenna element shown in Figure 4 and in Figure 7, respectively, the transmitter array and receiver array can be configured as in Figure 12. While the arrays configurations of the traditional TI’ LFMCW radar introduced in [30,31] are shown in Figure 13. Figure 14a,b illustrate the radiation patterns of the transmitter and receiver arrays of the proposed VAA, respectively, while Figure 14c,d show the patterns of the transmitter and receiver arrays of the TI’ LFMCW radar, respectively.

**Figure 15:**
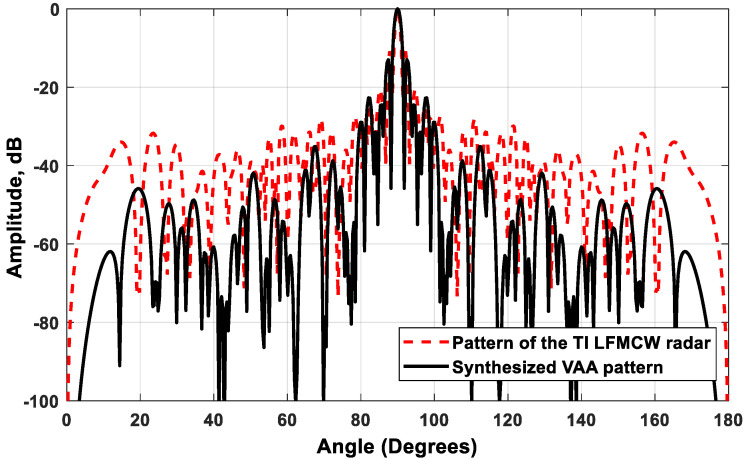
Radiation pattern of the proposed VAA compared to the traditional TI’ LFMCW radar pattern.

A comparison between the synthesized VAA pattern and the pattern of the traditional TI’ LFMCW radar is shown in Figure 15. It is clear that the two patterns had the same HPBW = $1.51^{{}^{\circ}}$, while the proposed VAA pattern had a faster-decaying SLL than that of the TI’ LFMCW pattern. The synthesized VAA had ${\mathrm{SLL}}_{max}=-13\;\mathrm{dB}$ and ${\mathrm{SLL}}_{min}=-74.78\;\mathrm{dB},$ while the TI’ LFMCW array had ${\mathrm{SLL}}_{max}=-10.88\;\mathrm{dB}$ and ${\mathrm{SLL}}_{min}=-41.4\;\mathrm{dB}$. The gain of the proposed VAA approached 47.2 dBi, while that of the TI’ LFMCW approached 26 dBi. The area of the TI’ LFMCW approached $613{\;\lambda }^{2}$, while the area of the proposed VAA approached $337{\;\lambda }^{2}$, which saved the required area by 45%. It is clear that the proposed VAA configuration was superior to the TI’ LFMCW configuration in terms of the implementation area, SLL, and realized array gain. It is worth noting that the saved area could be translated into achieving multiples of the array gain, and thus increased the radar detection range.

C. Experimental results

**Figure 16:**
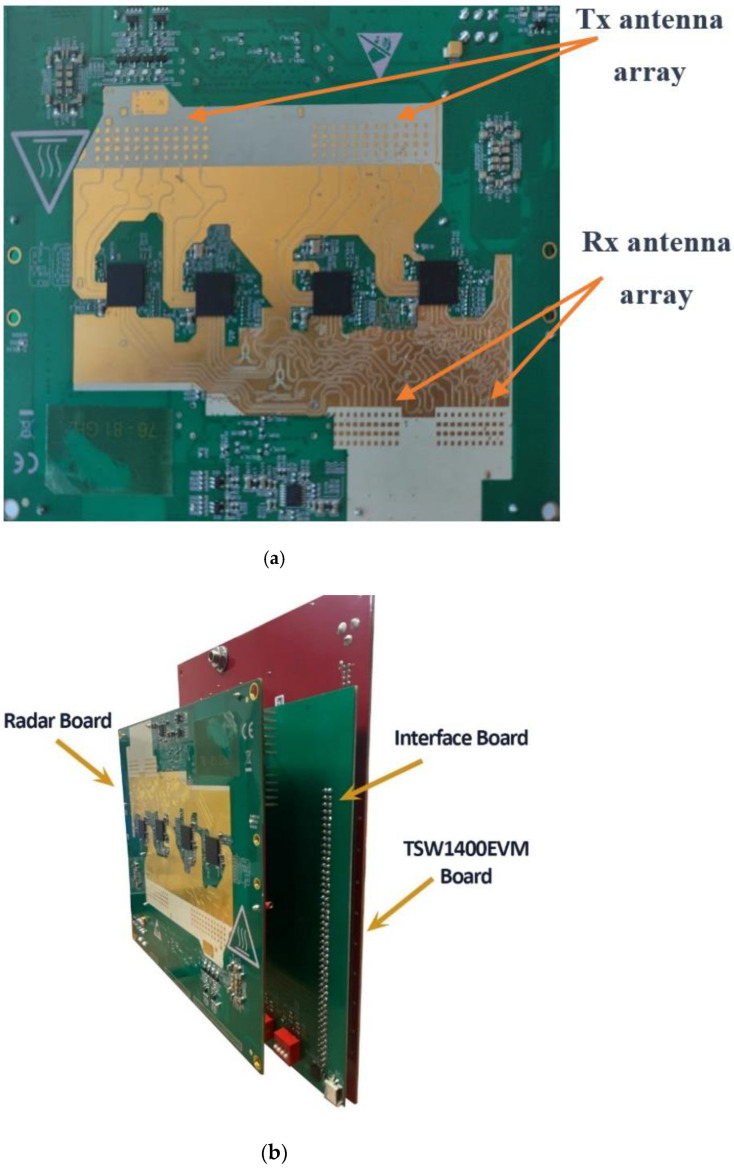
77 GHz radar system: (**a**) fabricated VAA radar board; (**b**) fabricated VAA radar board connected to the TI’ TSW1400EVM board via an interface board.

In order to test the proposed high-gain and low-SLL VAA configuration, the proposed VAA was integrated in a working radar system, as shown in Figure 16. This radar system was designed and fabricated using low-power 45 nm Texas Instruments (TI) RFCMOS technology with the integration of four AWR1243 RF chips, as in the case of the traditional TI’ LFMCW radar system [31]. Therefore, we had a limited number of transmitting sources, which limited the number of transmitter antenna elements to only 12. By following our design criteria introduced in Section 4.2, we introduced the complete radar system shown in Figure 16a. The radar system had two main boards: the fabricated VAA antenna board shown in Figure 16a, and the TSW1400EVM board from Texas Instruments; the two boards were connected via an interface board, as shown in Figure 16b. The TSW1400EVM board was used to upload firmware to the RF chips through the UART protocol, and to send configuration messages through the SPI protocol. These messages configured radar parameters like bandwidth, slope, PRF, data rate, data format, and sampling rate.

**Figure 17:**
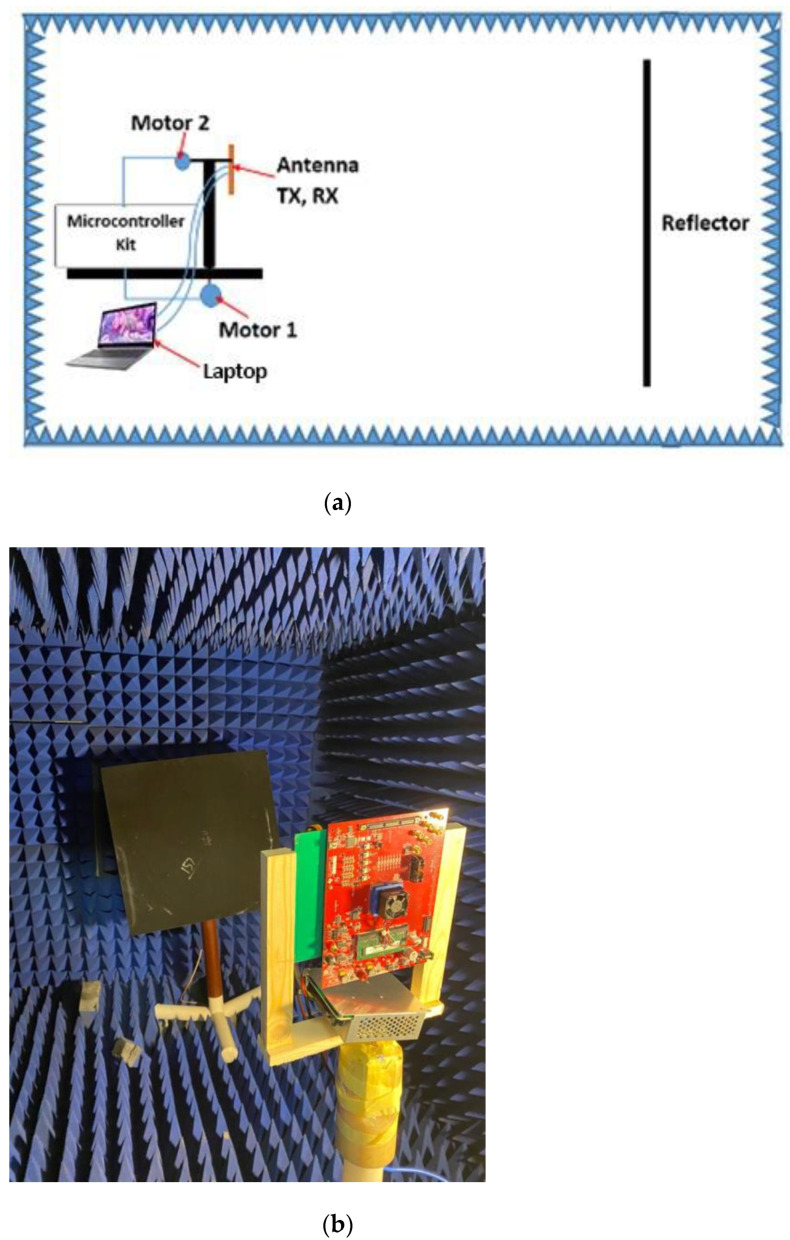
Setup system for VAA measurements: (**a**) proposed measurement system; (**b**) photo inside anechoic chamber.

**Figure 18:**
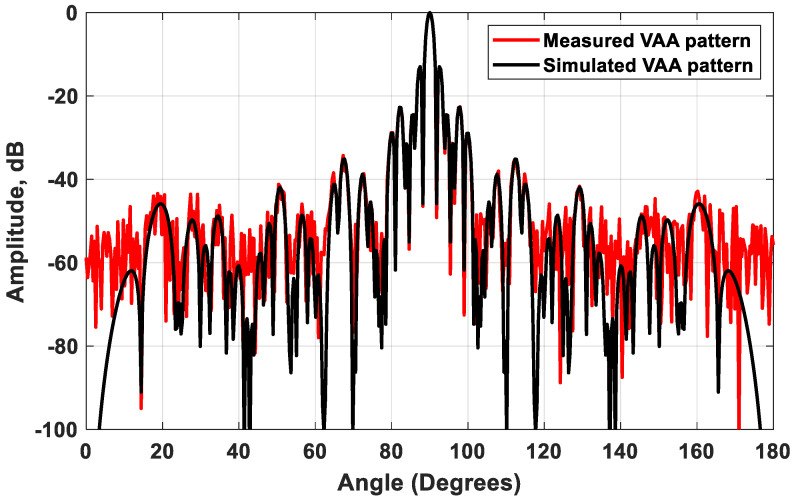
A comparison between the measured and simulated VAA patterns.

The setup for the measurement of the VAA radiation pattern is illustrated in Figure 17. Figure 17a explains the schematic diagram of the proposed measurement setup, while Figure 17b illustrates a photo for the practical setup. The device under test (DUT) was mounted on a Teflon rod that could be rotated in the azimuth direction. The DUT was fixed on top of this Teflon rod on another vertically rotated Teflon rod to provide the radiation pattern measurements for the azimuth and elevation planes. The fabricated radar system was connected to a laptop for controlling the transmitting function of the radar and for capturing the backscattered signal. The backscattered signal was recorded at different angles, and the normalized radiation pattern was plotted compared to the simulated results, as illustrated in Figure 18. It was noticed that the measured and simulated radiation patterns were highly matched; the two patterns had the same HPBW = $1.51^{{}^{\circ}}$ and the same maximum side lobe level ${\mathrm{SLL}}_{max}=-13\;\mathrm{dB}$; while the minimum SLLs of the measured and simulated patterns were around −55 dB and −74.78 dB, respectively.

## 5. Conclusions

A novel flexible scheme for synthesizing a VAA system for automotive radar systems was introduced. The proposed scheme aimed to reduce the size, minimize the SLL, and increase the gain in a cost-effective procedure. The scheme allowed for different VAA configurations that took into consideration the available area, number of transmitters, and number of receivers. The scheme was applied to the design of a practical automotive radar with 12 transmitters and 16 receivers. The proposed VAA distribution saved the required implementation area by 45% compared to the TI’ LFMCW antenna array. The VAA system was fabricated and tested in an anechoic chamber, and it was noticed that the measured and simulated radiation patterns highly coincided; the two patterns had the same HPBW =$\;1.51^{{}^{\circ}\;}$ and the same maximum side lobe level ${\mathrm{SLL}}_{max}=-13\;\mathrm{dB}$, while the minimum SLLs of the measured and simulated patterns were around −55 dB and −74.78 dB, respectively. The realized VAA achieved a gain of 47.2 dBi, which was much higher than that of the TI’ LFMCW radar, which equaled 26 dBi.

## Figures and Tables

**Table 1:** The parameters of the proposed transmitter antenna elements.

Parameter	Value in (mm)	Parameter	Value in (mm)
g	1.5918	P2	0.88763
Wf	0.1893	P3	0.923
Wg	0.1056	P4	0.12424
Lf	0.09016	Lf2	1.1092
Wf1	0.0888076	S1	2.517
P1	0.9584		

## Data Availability

Not applicable.
